# Combined uterine injury and high rectal perforation complicating abdominal ectopic pregnancy: A case report

**DOI:** 10.1016/j.ijscr.2024.109823

**Published:** 2024-05-29

**Authors:** Bacem Zaidi, Wael Gazzah, Alaeddine Ben Amor, Sihem Sindi, Walid Maraach, Zied Mensi

**Affiliations:** a University of Sousse, Faculty of Medicine, Department of Surgery, Ibn El Jazzar Hospital, Kairouan, Tunisia; b University of Sousse, Faculty of Medicine, Department of Urology, Ibn El Jazzar Hospital, Kairouan, Tunisia; c University of Sousse, Faculty of Medicine, Department of Orthopedic Surgery, Ibn El Jazzar Hospital, Kairouan, Tunisia

**Keywords:** Abdominal pregnancy, Uterine perforation, Rectal perforation, Ectopic pregnancy, Case report

## Abstract

**Introduction and importance:**

Abdominal pregnancy is a rare and potentially fatal variant of ectopic pregnancy, presenting unique clinical challenges. This report discusses an unusual case of abdominal pregnancy associated with uterine and high rectal perforations, complications that are rarely reported in clinical practice.

**Case presentation:**

We report a case involving a 31-year-old woman from a rural area, with a psychiatric history, presenting severe abdominal pain, vomiting, and constipation. Initial investigations revealed a hemopneumoperitoneum and a fetal skeleton in the pelvic area by CT, leading to a diagnosis of abdominal pregnancy. Surgical findings included a nonviable fetus, approximately 5 months gestational age, and perforations in both the rectum and the posterior uterine wall.

**Clinical discussion:**

The patient underwent extensive surgery, including placental dissection, anterior rectal resection, Hartmann's colostomy, hysterorrhaphy, and drainage of the peritoneal cavity. The complexity of managing abdominal pregnancy, especially with additional complications such as organ perforations, poses significant surgical challenges. This case emphasizes the need to consider abdominal pregnancy in differential diagnoses of abdominal pain in women, due to the risk of misdiagnosis and complex surgical requirements.

**Conclusion:**

This case highlights the critical importance of prompt diagnosis and comprehensive care in managing rare and life-threatening presentations of abdominal pregnancy. It underscores the need to increase awareness among clinicians for timely intervention and provides information on the complexities of surgical management in cases with additional organ perforations.

## Highlights

• Rare case of abdominal ectopic pregnancy with concurrent uterine and high rectal perforations
• Successful management of a complex surgical scenario in a patient with psychiatric history
• Utilization of advanced imaging for accurate diagnosis of an intricate abdominal pregnancy
• Comprehensive surgical intervention including placental dissection, anterior rectal resection, and Hartmann's colostomy
• Highlights the need for differential diagnosis awareness and surgical adaptability in rare ectopic pregnancies

## 1 Introduction

Abdominal pregnancy is a rare and extraordinary variant of ectopic pregnancies, which occurs when the embryo implants within the peritoneal cavity, specifically outside the ovary, fallopian tube, and broad ligament. This condition is estimated to manifest itself in approximately 1 in 10,000 to 30,000 pregnancies [1,2]. Unlike more common ectopic pregnancies, abdominal pregnancies pose severe risks due to the likelihood of massive hemorrhage, with this risk predominantly arising from the placenta, which can detach partially or completely at any stage during pregnancy [3]. Maternal mortality rates for abdominal pregnancies range from 0.5 to 18 %, with perinatal mortality rates between 40 and 95 %, significantly higher than at other ectopic sites [4,5]. Ectopic pregnancies occur at other locations 7.7 times more frequently, whereas intrauterine pregnancies are 90 times more common [3].

Diagnosing an abdominal pregnancy presents significant challenges. These pregnancies often go unnoticed until they reach an advanced stage, in stark contrast to the more easily detected tubal ectopic pregnancies. This delay in diagnosis significantly contributes to the high rates of maternal complications associated with abdominal pregnancies [6].

The primary management strategy for abdominal pregnancy is surgical intervention. However, removal of the ectopic pregnancy mass involves substantial risks, including intractable bleeding and potential organ damage. These risks are largely due to the deep infiltration of trophoblastic tissue into adjacent organs and structures [7].

This report presents a complex case of abdominal pregnancy, initially considered under multiple differential diagnoses, highlighting the diagnostic complexities and the critical need for increased clinical awareness and timely intervention.

## 2 Presentation of the case

A 31-year-old woman, gravida 2, para 1, residing in a rural area and with a known psychiatric condition, specifically bipolar disorder, was taken to the emergency department with severe abdominal pain, episodes of vomiting, and constipation. The psychiatric diagnosis had been managed with medication, which may have influenced her initial refusal to acknowledge symptoms suggestive of pregnancy.

Upon physical examination, the patient appeared pale and visibly distressed. Clinical findings included a fever of 38.5 °C, and a heart rate of 100 beats per minute, which, while at the upper limit of normal, suggested a stress response. Her blood pressure was 110/60 mmHg, considered indicative of relative hypotension given her clinical state of potential hypovolemic shock due to internal bleeding.

Abdominal examination revealed distention and palpation tenderness, indicative of significant intraabdominal pathology. Laboratory investigations after admission showed a hemoglobin level of 11 g/dl, indicating mild anemia possibly due to chronic disease or acute blood loss. The white blood cell count was 4400 cells/μl, and the platelet count was 232,000/μl, both within normal limits but suggestive of an acute stress response.

**Fig. 1:**
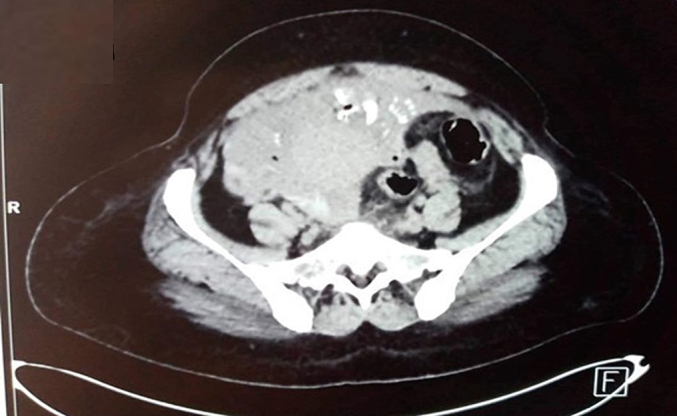
Detailed view of hemopneumoperitoneum – This image shows the significant presence of hemopneumoperitoneum revealed by abdominal CT, illustrating the extent of intraperitoneal air and fluid, indicative of a complex abdominal pregnancy.

**Fig. 2:**
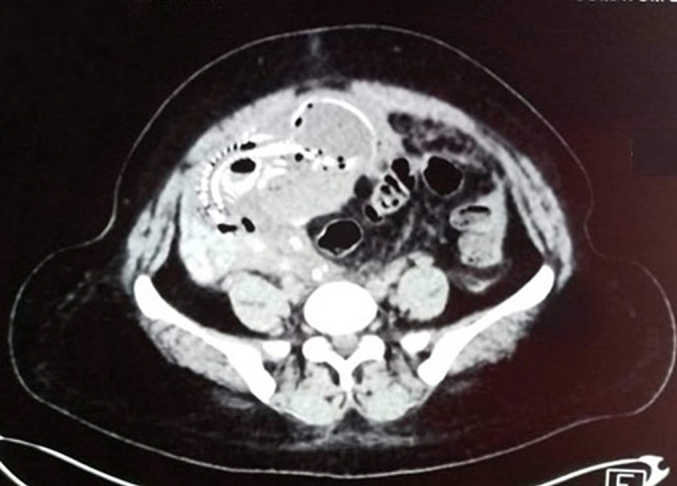
Identification of the fetal bone skeleton in the pelvic area - Captured by abdominal CT, this figure highlights the fetal bony structure located within the pelvic region, a key diagnostic feature supporting the identification of an advanced abdominal pregnancy.

An abdominal CT scan demonstrated extensive hemopneumoperitoneum (Fig. 1), revealing a significant presence of air and fluid in the peritoneal cavity indicative of organ perforation. Furthermore, the scan identified the presence of a bony fetal skeleton in the pelvic region (Fig. 2), crucial to diagnosing abdominal pregnancy. The fetal remains and associated findings led to an urgent surgical intervention.

The initial evaluations were thorough, but the patient initially resisted acknowledging symptoms suggestive of pregnancy. This hesitation could be attributed to psychological factors associated with her bipolar disorder, potentially compounded by the stigma associated with her psychiatric condition.

Intravenous fluid therapy was administered for stabilization and blood samples were collected for type and cross-match purposes, to prepare for the potential need for transfusion during the surgical intervention. After obtaining written and informed consent from the patient, she was promptly taken to the operating room.

**Fig. 3:**
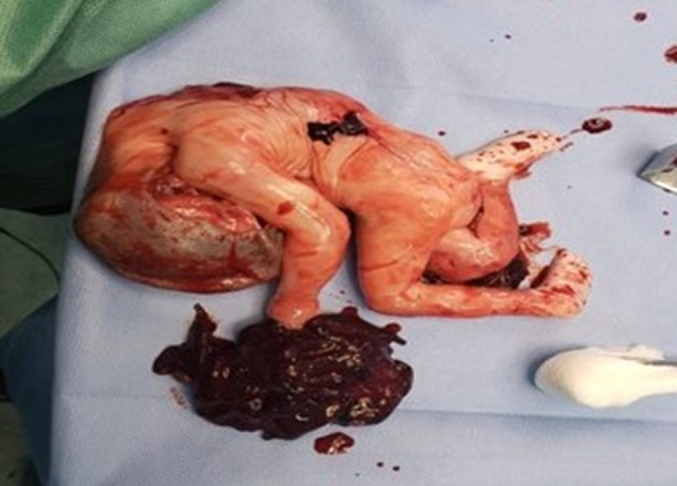
Intraoperative discovery of the nonviable fetus - This image depicts the nonviable fetus encountered during surgery, with a notable absence of the right upper limb, alongside the partially fragmented placenta.

During the laparotomy, significant findings were encountered. There was extensive hemoperitoneum with an estimated blood loss of approximately 2 l, indicative of major internal bleeding. The surgery revealed a nonviable fetus approximately 5 months gestational age, missing its right upper limb, which weighed 600 g and measured 20 cm in length. Adjacent to the fetus, a partially fragmented placenta was discovered, attached to the posterior wall of the uterus (Fig. 3), highlighting the complexity and unusual presentation of abdominal pregnancy.

The complexity of the case was further compounded by the discovery of a 3 mm perforation in the high rectum, which had allowed fecal matter into the peritoneal cavity, suggesting the onset of stercoral peritonitis. Furthermore, a large 5 cm perforation was found in the posterior wall of the uterus, which presents significant surgical challenges and requires complex reparative procedures.

To address the complexities of this case, an extensive surgical procedure was required. The operation involved meticulous dissection of the placenta from the posterior wall of the uterus, due to its abnormal implantation and extensive infiltration. An anterior rectum resection was performed to effectively manage rectal perforation, followed by the formation of a Hartmann colostomy on the left side to temporarily divert fecal matter and allow healing of rectal tissue. Furthermore, hysterorrhaphy was performed to repair the significant uterine perforation. The procedure was completed with thorough abdominal irrigation using a saline solution to minimize the risk of infection, and adequate drainage of the peritoneal cavity was established to prevent fluid accumulation.

Postoperatively, the patient demonstrated stable recovery, which negated the need for blood transfusions. The patient was closely monitored and her hemoglobin levels remained stable, indicating effective treatment of potential hemorrhagic complications. She was discharged in good condition after a 5-day hospital stay, which is indicative of a successful initial recovery phase. During follow-up visits two weeks after discharge, it was observed that recovery was uneventful. She reported no complaints and clinical evaluations did not reveal any abnormalities. The patient was scheduled to undergo a procedure to restore digestive continuity two months after the initial surgery, with the aim of reversing the colostomy and improving her quality of life.

## 3 Discussion

Abdominal pregnancies can be classified into two distinct categories. The first, known as primary abdominal pregnancy, involves implantation of the fertilized ovum directly into the abdominal cavity, while the fallopian tubes and ovaries remain uninvolved. Primary abdominal pregnancies are extremely rare, with only a handful of cases documented [8]. In contrast, most advanced extrauterine pregnancies fall under the category of secondary abdominal pregnancy, where initially an extrauterine tubal pregnancy ruptures or aborts, and then reimplants within the abdominal cavity.

Abdominal pain is the predominant symptom in abdominal pregnancies, but diagnosis during the antenatal period is challenging, with only approximately 45 % of cases identified before birth. This difficulty often leads to misdiagnosis, as was the case in our report, where the condition was initially mistaken for an intrauterine pregnancy [9].

The complexity of achieving a preoperative diagnosis stems from the polymorphic clinical presentation and nonspecific nature of the pain. Since each location of pregnancy exhibits unique semiological characteristics, abdominal pain in such cases often presents atypically due to organ displacement during pregnancy, further complicating the timely diagnosis [10,11]. Emergent abdominal conditions such as hemoperitoneum, peritonitis, or acute intestinal obstruction can sometimes be the initial indicators of an abdominal pregnancy [12,13]. In the case we presented, the patient, possibly due to lack of awareness or intentional concealment arising from her psychiatric history, did not recognize her pregnancy and the diagnosis was ultimately established by CT imaging.

In approximately half of the cases, preoperative suspicion of abdominal pregnancy is raised when an empty uterus is observed in conjunction with a gestational sac or a distinct mass separate from the uterus, adnexa, and ovaries. Advanced imaging techniques such as CT and magnetic resonance imaging are instrumental in confirming the diagnosis, elucidating anatomical relationships, discerning possible vascular connections, and evaluating the degree of placental adhesion [14]. In the case of our patient, given the absence of visible pregnancy indicators and the clinical presentation, a CT scan was performed. This scan revealed a complex abdominal pregnancy, prompting the decision to proceed with a laparotomy.

The consensus among medical professionals is that exploratory laparotomy is the preferred approach to surgical management once an abdominal pregnancy is definitively diagnosed. This procedure often presents unexpected challenges, particularly in achieving effective hemostasis. This complexity arises because the placenta often adheres to surrounding organs as it seeks vascularization, complicating decisions regarding its removal [15,16]. In the case of our patient, we immediately moved to surgical intervention following a brief period of preoperative preparation.

In situations where the placenta or a portion of it remains attached to the uterus, a hysterectomy may be necessary. Conversely, if the placenta implants on non-essential organs such as the omentum or a tube, it becomes crucial to remove both the organ and the placenta. The optimal strategy is to sever the umbilical cord as close to the placenta as possible, particularly if the placenta adheres to critical organs such as the colon, small intestine, or the anterior wall of the rectum [14,15].

Although surgical extraction of the fetus and opening of the sac in cases of abdominal pregnancy are generally straightforward, management of associated bleeding, adhesions, and, particularly, the placenta, often presents substantial challenges. The ideal approach involves complete removal of the sac, which includes the fetus, the membranes, and the placenta. However, during placental removal, surgeons may encounter severe bleeding that cannot be easily controlled using conventional methods such as packing, clamping, or suturing. Therefore, preparation for the management of excessive bleeding is crucial.

In situations where the placenta adheres to vital organs, such as the liver, spleen, or intestines, it is often advisable to leave it in situ. The detachment of the placenta from these structures can lead to unmanageable bleeding [17]. In our case, the patient underwent both hysterorrhaphy and Hartmann colostomy. These procedures were required by substantial perforations located in the posterior wall of the uterus and in the upper region of the rectum.

This case report of abdominal pregnancy with associated uterine and rectal perforations has several key clinical implications. It emphasizes the need for greater vigilance in diagnosing ectopic pregnancies, especially in patients with nonspecific symptoms such as abdominal pain. The complex surgical challenges highlighted in this case underscore the importance of preparation and adaptability in the management of abdominal pregnancies with additional complications. This report contributes significantly to the existing literature by describing a rare clinical scenario, thus enriching the knowledge base of clinicians and researchers. This underscores the impact of timely and accurate diagnosis on patient outcomes and reinforces the need for further research to improve diagnostic tools and surgical techniques for complex ectopic pregnancies. The findings of this case open new avenues for future research, particularly in the realm of early detection strategies and understanding risk factors for abdominal pregnancies, which is a crucial step in the advancement of obstetric and gynecological practices.

## 4 Conclusion

Abdominal pregnancy is one of the most severe and rare forms of extrauterine pregnancy. This requires a focused approach to the criteria for diagnosis, choice and timing of treatment strategies, perioperative management, and diligent postoperative follow-up. Clinicians must improve their understanding and skills to facilitate early detection and minimize patient risks and complications.

Misdiagnosis of abdominal pregnancies has a significant potential for adverse outcomes. Therefore, increased vigilance coupled with an improved understanding and interpretation of both clinical presentations and imaging findings is imperative for all medical professionals involved in the care of these patients.

There is a broad consensus in the medical literature on the necessity of surgical intervention, specifically exploratory laparotomy, in the management of abdominal pregnancies. This approach is crucial for providing optimal patient care and mitigating the possibility of future complications, although it may present unforeseen challenges during the surgical process.

## Ethical approval

This study is exempt from ethical approval as per the policies of Ibn El Jazzar Hospital.

## Funding

None.

## Author contribution

All authors have contributed equally to the work reported in this manuscript, including the conception, design, execution, data acquisition, analysis and interpretation, and the drafting and revising of the manuscript for important intellectual content.

## Guarantor

Wael Gazzah

## Patient perspective

The patient expressed relief and satisfaction with the treatments received, highlighting their effectiveness and the care provided by the medical team.

## Informed consent

Written informed consent was obtained from the patient for the publication of clinical details and/or clinical images. A copy of the consent form is available for review by the editor of this journal and will be provided upon request. The consent form used followed the guidelines of the Hindawi consent to publication form.

## SCARE guidelines

This study was reported in line with the SCARE criteria [18].

## Conflict of interest statement

The authors declare no conflicts of interest regarding the publication of this article.
